# Maize feedstocks with improved digestibility reduce the costs and environmental impacts of biomass pretreatment and saccharification

**DOI:** 10.1186/s13068-016-0479-0

**Published:** 2016-03-15

**Authors:** Andres F. Torres, Petronella M. Slegers, Cornelie M. M. Noordam-Boot, Oene Dolstra, Louis Vlaswinkel, Anton J. B. van Boxtel, Richard G. F. Visser, Luisa M. Trindade

**Affiliations:** Wageningen UR Plant Breeding, Wageningen University and Research Centre, P.O. Box 386, 6700 AJ Wageningen, The Netherlands; Plant Biotechnology Laboratory (COCIBA), Universidad San Francisco de Quito USFQ, Diego de Robles y Vía Interoceánica, Cumbayá, Ecuador; Biobased Chemistry and Technology, Wageningen University and Research Centre, P.O. Box 17, 6700 AA Wageningen, The Netherlands; Limagrain Nederland B.V., Rilland, The Netherlands

**Keywords:** Maize, Cell wall digestibility, Biomass yield, Technoeconomic, Refinery, Pretreatment

## Abstract

**Background:**

Despite the recognition that feedstock composition influences biomass conversion efficiency, limited information exists as to how bioenergy crops with reduced recalcitrance can improve the economics and sustainability of cellulosic fuel conversion platforms. We have compared the bioenergy potential—estimated as total glucose productivity per hectare (TGP)—of maize cultivars contrasting for cell wall digestibility across processing conditions of increasing thermochemical severity. In addition, exploratory environmental impact and economic modeling were used to assess whether the development of bioenergy feedstocks with improved cell wall digestibility can enhance the environmental performance and reduce the costs of biomass pretreatment and enzymatic conversion.

**Results:**

Systematic genetic gains in cell wall degradability can lead to significant advances in the productivity (TGP) of cellulosic fuel biorefineries under low severity processing; only if gains in digestibility are not accompanied by substantial yield penalties. For a hypothetical maize genotype combining the best characteristics available in the evaluated cultivar panel, TGP under mild processing conditions (~3.7 t ha^−1^) matched the highest realizable yields possible at the highest processing severity. Under this scenario, both, the environmental impacts and processing costs for the pretreatment and enzymatic saccharification of maize stover were reduced by 15 %, given lower chemical and heat consumption.

**Conclusions:**

Genetic improvements in cell wall composition leading to superior cell wall digestibility can be advantageous for cellulosic fuel production, especially if “less severe” processing regimes are favored for further development. Exploratory results indicate potential cost and environmental impact reductions for the pretreatment and enzymatic saccharification of maize feedstocks exhibiting higher cell wall degradability. Conceptually, these results demonstrate that the advance of bioenergy cultivars with improved biomass degradability can enhance the performance of currently available biomass-to-ethanol conversion systems.

**Electronic supplementary material:**

The online version of this article (doi:10.1186/s13068-016-0479-0) contains supplementary material, which is available to authorized users.

## Background

Within the domain of cellulosic fuel research, major efforts have been devoted towards the development of advanced lignocellulosic crops designed to meet the demands of the industry. Plant breeders are now faced with the challenge of identifying highly productive biomass varieties which can be produced inexpensively, sustainably, and in abundant quantities [1]. Furthermore, since lignocellulose recalcitrance is a critical barrier towards the efficient conversion of plant biomass into biofuels and biomaterials [2, 3], improving the processing amenability of lignocellulosic crops remains a pivotal goal of bioenergy crop breeding endeavors [1].

Our understanding of the composition, structure, and biosynthesis of the plant cell wall has expanded greatly in the last decade. This knowledge has enabled the development of breeding strategies targeting the modification of key cell wall compositional features that can reduce the inherent recalcitrance of lignocellulosic substrates. Extensive evidence suggests that it is possible to advance lignocellulosic crops requiring lower energetic and chemical inputs for their effective fractionation into fermentable monosaccharides [4–8].

Despite the prevalent notion that biomass composition can exert a determinant influence on biomass-to-biofuel conversion efficiency, limited information exists as to how bioenergy crops with reduced lignocellulose recalcitrance can improve the economics and environmental performance of the industry. To date, techno-economic and life cycle assessments of cellulosic fuel refineries have minimized the role of biomass feedstocks to cost, productivity, and availability considerations [9–14]. These comparative analyses often imply that the profitability and sustainability of cellulosic fuels can be primarily attained through innovations in process engineering or advances in the yield productivity (per hectare) of biomass species. Under these provisions, advancing lignocellulosic crops with increased cell wall degradability could prove detrimental to the industry, since alterations in cell wall composition may lead to concomitant reductions (albeit, of varying degrees) in yield productivity [15].

With an ongoing debate as to whether bioenergy crop breeding endeavors should focus exclusively on improving biomass yield performance, the main objective of this study was to conceptually demonstrate how, and under which circumstances, bioenergy crops with improved cell wall degradability can enhance the environmental and economic performance of biomass-to-ethanol conversion processes. To this end, we have analyzed the bioenergy potential—in relation to yield of total fermentable glucose per hectare—of a set of forage maize commercial cultivars contrasting for ruminal cell wall digestibility across a range of processing conditions of increasing thermochemical severity. A focus on the relationship between biomass yield and processing amenability has been warranted, as general convention dictates that yield penalties are a common consequence of breeding efforts leading to reduced lignocellulose recalcitrance. In addition, explorative economic and environmental impact modeling focused on the pretreatment process were used to assess whether the development of bioenergy feedstocks with improved cell wall digestibility can improve the sustainability and cost performance of biomass thermochemical processing and saccharification. Our study focused on the production of cellulosic ethanol derived via dilute-acid pretreatment and enzymatic hydrolysis as the latter constitutes the most advanced and commercially represented platform in the industry [16]. Accordingly, we used maize as a “model” biomass feedstock because of the extensive availability and accessibility of highly productive commercial cultivars showcasing extensive variation for biomass compositional quality and cell wall degradability [1, 8].

## Results and discussion

### Commercial forage maize cultivars display substantial diversity in cell wall composition and cell wall digestibility

Entries evaluated in this study comprised forage maize cultivars bred for Northern-European markets. The panel displayed a broad range of variation for in vitro ruminal cell wall digestibility (CWD) with a maximal difference between entries of nearly 25 % units (Table 1). Henceforth, all commercial cultivars were classified based on their CWD ratings as either having “Excellent,” “Good”, or “Poor” cell wall digestibility. The counterparts of five proprietary hybrids carrying either the brown-midrib 3 (*bm3*) or a biogemma proprietary [17] brown-midrib 1 mutation (*bm1*) were cataloged as “Cell Wall Mutants”.

**Table 1:** Whole-plant biomass yield (in t ha^−1^) and digestibility rating of Northern-European maize silage cultivars and experimental hybrids

Accession	Yield (t ha^−1^)	CWD^a^ (%)	Digestibility class
HYB-001	18.7	36.6	Excellent
HYB-002	21.1	34.1	Excellent
HYB-003	20.3	33.8	Excellent
HYB-004	19.7	30.7	Excellent
HYB-005	18.5	34.1	Excellent
HYB-006	19.3	28.1	Good
HYB-007	19.4	24.8	Good
HYB-008	18.4	24.3	Good
HYB-009	19.0	25.0	Good
HYB-010	20.5	28.4	Good
HYB-011	17.3	29.3	Good
HYB-012	20.8	15.7	Poor
HYB-013	20.8	19.9	Poor
HYB-014	21.0	14.2	Poor
HYB-015	20.5	18.8	Poor
HYB-016	21.8	17.2	Poor
HYB-017	20.4	15.9	Poor
HYB-018	20.6	15.2	Poor
HYB-010-*bm3*	17.8	38.8	Cell wall mutant
HYB-011-*bm3*	15.2	35.0	Cell wall mutant
HYB-006-*bm1*	15.8	33.2	Cell wall mutant
HYB-009-*bm1*	16.8	34.3	Cell wall mutant
HYB-014-*bm1*	16.5	26.0	Cell wall mutant
Mean	19.3	26.7	
F probability^b^	***	***	
L.S.D^c^	1.6	5.7	

^a^CWD: In vitro cell wall digestibility determined as the difference in neutral detergent fiber content before and after sample incubation in rumen liquor for 48 h relative to neutral detergent fiber content prior to incubation

^b^Significance of differences between entries as determined by ANOVA; *p* < 0.05 (*), *p* < 0.01(**), and *p* < 0.001(***), NS indicates non-significant differences

^c^Least significant differences of means (5 % level)

**Fig. 1:**
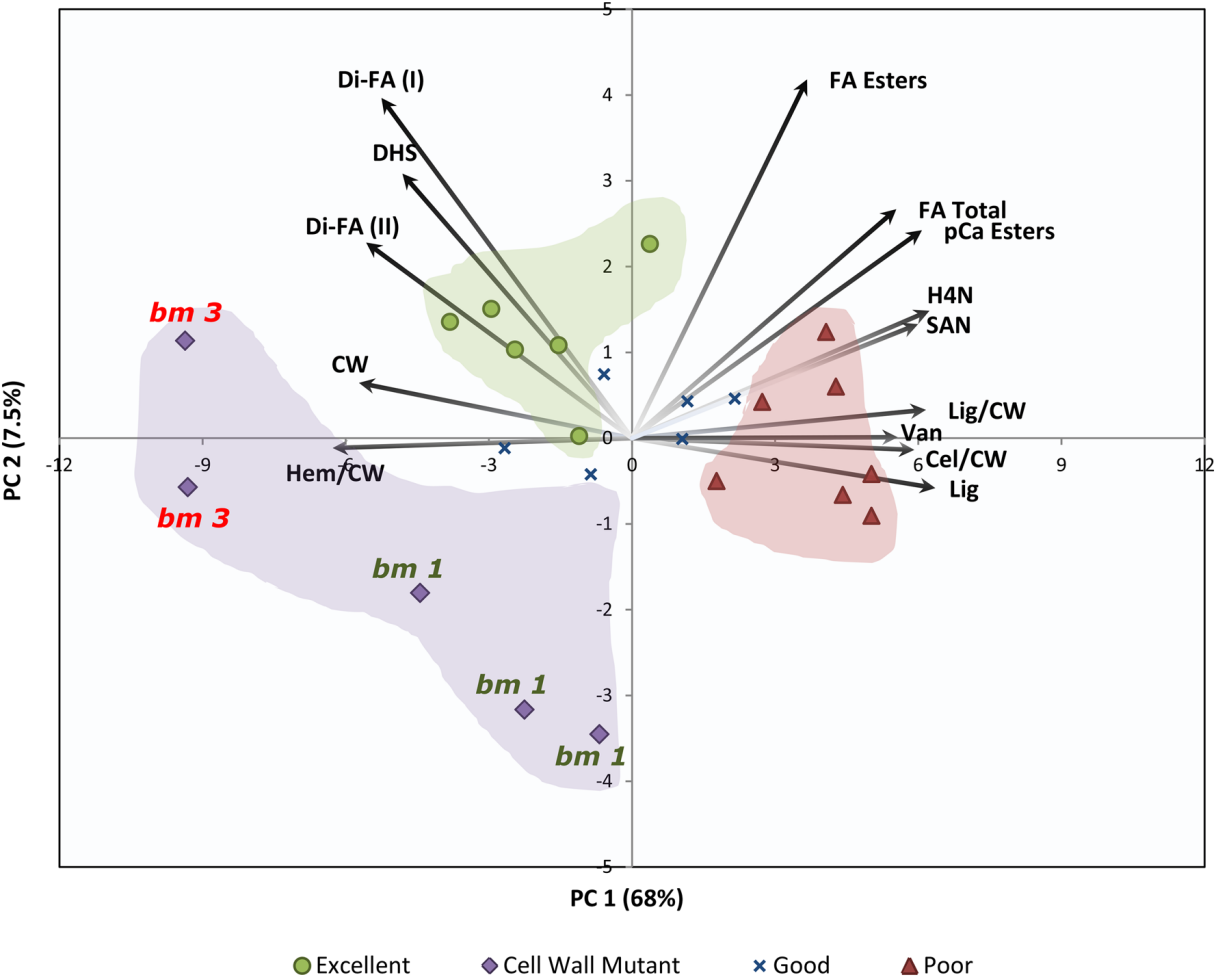
Principal component biplot displaying the classification of a panel of Northern-European forage maize cultivars based on stem fiber and cell wall components. Cultivars were classified based on their DINAG ratings as either having “Excellent” (*Green*), “Good” (*Blue*), or “Poor” (*Red*) cell wall digestibility. The five proprietary hybrids carrying either the *bm3* or *bm1* mutations were cataloged as “Cell Wall Mutants” (*Purple*). *Black vectors* summarize the correlation between relevant feedstock compositional characters and the corresponding principal component

Highly significant (*p* < 0.001) differences were detected for all investigated cell wall traits (Table 2). Clear distinctions could also be made between the cell wall compositional profiles of the four distinct cultivar classes (Fig. 1). Multivariate analysis reveals that compositional diversity observed across entries could be primarily ascribed to variation in the phenolic and hemicellulosic fractions of their cell walls (PC 1 = 68 %). A direct comparison between the “Excellent” and “Poor” cultivar classes confirms that (breeder’s) selection for enhanced ruminal cell wall digestibility has favored cell walls with reduced lignin content and increased hemicellulose concentration [18–25]. Cultivars with high CWD ratings were also found to have cell walls with a higher concentration of di-ferulic esters, as well as an increased degree of hemicellulose substitution (measured as the ratio of cell wall arabinose to xylose); the latter presumed indicative of the degree of side-chain glycosylation of glucoronoarabinoxylan. In conjunction, a higher concentration of di-ferulic esters and a higher degree of glucoronoarabinoxylan side-chain glycosylation could imply an increased incidence of hemicellulose-to-hemicellulose cross-linking in the cell walls of highly digestible entries [26–28]. In our view, maize cell walls with reduced lignin content can restructure their hydrophobic cell wall matrix by increasing the concentration and rate of cross-linking of glucoronoarabinoxylan molecules to maintain the physical integrity of the cell wall. Incidentally, highly branched glucoronoarabinoxylan polymers (deemed necessary for a greater extent of cross-linking) exhibit reduced adsorption affinity to cellulose and improved water solubility, and have been shown to significantly improve the enzymatic depolymerization of maize cell walls [7].

**Table 2:** Comparison of cell wall compositional profiles for a panel of commercial silage maize cultivars and experimental mutant counterparts of five cultivars

	CW	Cel	Lig	Cel/CW	Hem/CW	Lig/CW	pCa I	pCa II	FA I	FA II	Di-FA I	Di-FA II	DHS	H	S	G
HYB-001	613	363	54	574	338	88	25.9	24.4	7.67	9.73	0.08	0.16	0.13	3.43	15.3	10.7
HYB-002	566	330	52	562	347	92	25.8	24.1	7.68	9.76	0.07	0.12	0.12	3.34	15.5	11.9
HYB-003	675	373	70	579	318	103	28.4	26.4	8.39	10.86	0.07	0.12	0.12	3.85	15.2	12.0
HYB-004	631	374	62	578	324	98	27.3	25.7	7.48	9.81	0.05	0.11	0.13	3.74	16.7	12.3
HYB-005	598	351	55	570	339	91	26.2	24.1	8.22	10.35	0.06	0.14	0.12	3.55	14.7	11.5
HYB-006	682	409	68	587	312	100	27.1	25.0	8.31	10.70	0.04	0.11	0.11	3.80	15.1	11.5
HYB-007	693	401	79	582	305	113	29.8	28.1	7.92	10.72	0.05	0.12	0.12	4.20	16.8	13.4
HYB-008	632	411	64	575	324	101	26.6	24.9	7.97	10.26	0.06	0.13	0.12	3.47	15.4	11.2
HYB-009	672	410	72	590	304	106	29.5	27.3	8.16	10.70	0.04	0.11	0.11	4.20	16.8	12.7
HYB-010	630	378	63	583	318	99	26.9	25.0	7.57	10.0	0.04	0.12	0.12	3.68	16.3	12.2
HYB-011	657	387	64	572	331	97	24.1	22.6	7.20	9.85	0.06	0.17	0.13	3.28	14.1	11.6
HYB-012	717	445	85	590	291	119	32.6	30.5	7.94	10.89	0.02	0.09	0.11	4.73	18.6	14.2
HYB-013	678	419	75	598	283	119	28.9	27.1	7.81	10.38	0.04	0.13	0.12	3.88	17.1	12.1
HYB-014	704	435	81	594	292	114	32.1	30.0	7.83	10.71	0.01	0.08	0.10	4.8	18.2	15.1
HYB-015	699	435	76	598	288	114	28.6	26.7	7.78	10.42	0.04	0.11	0.12	3.99	16.4	13.2
HYB-016	715	432	85	585	306	109	32.7	30.9	7.78	10.73	0.02	0.07	0.11	4.46	19.0	13.5
HYB-017	705	435	78	597	293	110	32.6	30.4	8.48	11.31	0.05	0.10	0.11	4.52	16.4	12.9
HYB-018	714	438	81	592	294	114	32.5	30.5	8.05	11.01	0.03	0.10	0.11	4.89	18.9	14.1
HYB-010-*bm3*	625	339	41	555	377	66	17.4	15.8	7.57	10.0	0.09	0.19	0.14	2.45	6.9	9.6
HYB-011-*bm3*	638	353	46	560	368	72	17.8	16.7	7.44	9.58	0.10	0.18	0.13	2.56	6.5	10.0
HYB-006-*bm1*	690	399	61	573	340	88	19.7	17.9	7.57	9.73	0.04	0.11	0.11	2.80	11.6	10.2
HYB-009-*bm1*	684	388	58	563	353	85	17.9	16.5	7.43	9.59	0.06	0.15	0.12	2.57	10.7	10.1
HYB-014-*bm1*	678	397	64	580	326	94	23.0	21.1	7.52	9.89	0.03	0.08	0.10	3.37	13.1	12.4
F probability^a^	***	***	***	***	***	***	***	***	***	***	***	***	***	***	***	***
L.S.D.^b^	43	3	8	15	22	8	3.0	2.8	0.35	0.43	0.03	0.03	0.01	0.55	1.8	1.3

^a^Significance of differences between entries as determined by ANOVA; *p* < 0.05 (*), *p* < 0.01(**), and *p* < 0.001(***), NS indicates non-significant differences

^b^Least significant differences of means (5 % level)

Cell wall mutants also displayed good-to-excellent cell wall digestibility (Table 1), but in the principal component biplot these did not allocate with the other cultivar classes, nor did they form a resolute group (Fig. 1). Presumably, the latter reflects the contrasting genetic effects of the *bm3* and *bm1* mutations (Table 3). Relative to their hybrid counterpart, *bm3* mutants presented prominent reductions (~29 %) in lignin content, but also displayed statistically significant decrements in the concentration of p-coumaric acids (~31 %) and syringyl residues (~56 %). The *bm1* mutants displayed similar modification patterns in their cell wall phenolic profile, but decrements in lignin content (~17 %) and syringyl units (~30 %) were comparatively less profound. Moreover, relative to their hybrid counterpart, *bm1* mutants presented statistically significant reductions in the concentrations of cell wall esterified and total ferulic acids (~8 %). While improvements in the cell wall digestibility of *bm3* mutants can be ascribed to reductions in lignin content; higher digestibility in *bm1* mutants appeared to be a product of both, a decrease in lignin concentration and marked reductions in the extent of ferulate-mediated cross-linking between lignin polymers and (possibly) between lignin and hemicellulose.

**Table 3:** Targeted comparison of cell wall compositional profiles for five commercial maize cultivars and their corresponding cell wall mutant counterparts

	CW	Lig	Cel/CW	Hem/CW	Lig/CW	pCa I	pCa II	FA I	FA II	Di-FA I	Di-FA II	DHS	H	S	G
HYB-010	630	63	583	318	99	26.9	25.0	7.57	10.0	0.04	0.12	0.12	3.7	16.3	12.2
HYB-010-*bm3*	625	41*^,b^	555*	377*	66*	17.4*	15.8*	7.57	10.0	0.09*	0.19	0.14*	2.4*	6.9*	9.6
HYB-011	657	64	572	331	97	24.1	22.6	7.20	9.85	0.06	0.17	0.13	3.3	14.1	11.6
HYB-011-*bm3*	638	46*	560	368*	72*	17.8*	16.7*	7.44	9.58	0.10	0.18	0.13	2.6	6.5*	10.0
HYB-006	682	68	587	312	100	27.1	25.0	8.31	10.70	0.04	0.11	0.11	3.8	15.1	11.5
HYB-006-*bm1*	690	61	573	340	88	19.7*	17.9*	7.57*	9.73*	0.04	0.11	0.11	2.8*	11.6*	10.2
HYB-009	672	72	590	304	106	29.5	27.3	8.16	10.70	0.04	0.11	0.11	4.2	16.8	12.7
HYB-009-*bm1*	684	58	563*	353*	85*	17.9*	16.5*	7.43*	9.59*	0.06	0.15	0.12	2.6*	10.7*	10.1*
HYB-014	704	81	594	292	114	32.1	30.0	7.83	10.71	0.01	0.08	0.10	4.8	18.2	15.1
HYB-014-*bm1*	678	64*	580	326*	94*	23.0*	21.1*	7.52	9.89	0.03	0.08	0.10	3.4*	13.1*	12.4*
F probability^a^	***	***	***	***	***	***	***	***	***	***	***	***	***	***	***

^a^Significance of differences between entries as determined by ANOVA; *p* < 0.05 (*), *p* < 0.01(**), and *p* < 0.001(***), NS indicates non-significant differences

^b^For any specified stem fiber/cell wall component, “*” denotes a significant difference between an experimental mutant and its corresponding hybrid counterpart according to a Tukey HSD test (*P* = 0.05)

Ultimately, targeted reductions in lignin content will remain a key objective of efforts seeking to reduce the enzymatic recalcitrance of maize biomass, but our results confirm that improved cell wall digestibility can be attained through other mechanistic alterations of the plant cell wall. In this regard, Torres et al. [7] have shown that the accumulation of multiple beneficial compositional features will expectedly lead to the greatest gains in cell wall enzymatic convertibility in processing for cellulosic fuel. Therefore, the underlying genetic and biochemical foundations controlling the content, composition, and cross-linking of non-cellulosic cell wall polymers warrant further investigation, as these open unexplored avenues for the development of novel cell wall polymeric profiles with interesting projections for bio-based applications.

### Cultivars with high cell wall digestibility display improved glucose yields upon pretreatment and enzymatic saccharification

**Fig. 2:**
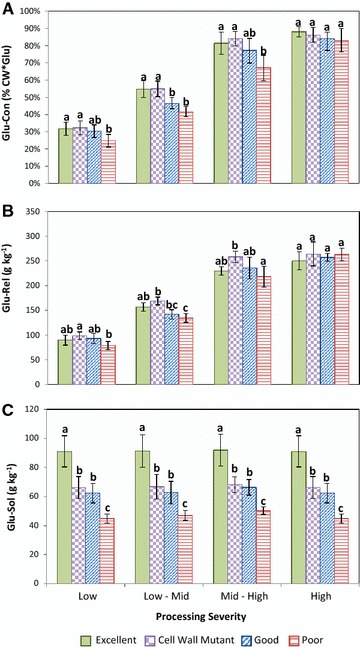
Conversion performance of four distinct cultivar classes (diverging in cell wall digestibility) for **A** Glu-Con, **B** Glu-Rel, and **C** Glu-Sol across pretreatments of increasing severity. Glu-Con is the percentage of total cell wall glucose released after pretreatment and enzymatic saccharification. Glu-Rel is the amount of glucose (g) released from 1 kg of dry biomass after pretreatment and enzymatic saccharification. Glu-Sol is the absolute amount of glucose (g) released from 1 kg of dry biomass into pretreatment liquors following thermochemical processing. Within each processing severity regime, similar letters above bars indicate non-significant differences according to a Tukey HSD test (*P* = 0.05)

The four cultivar classes showed statistically significant (*p* < 0.05) differences for bioconversion efficiency (Glu-Con) under nearly all examined pretreatment conditions; with the only exception ensuing at the harshest processing severity (Fig. 2A). The converged performance of all cultivar groups at highly stringent regimes was anticipated, given that under such conditions biomass conversion efficiency is primarily determined by the efficacy of the thermochemical process [7].

Figure 2A depicts the performance of the four cultivar classes for Glu-Con across the complete pretreatment series and demonstrates that the “Excellent” and “Cell Wall Mutant” classifications consistently outperformed classes displaying lower cell wall digestibility. From the onset of this investigation, we hypothesized that entries exhibiting improved forage quality would also display higher enzymatic convertibility upon thermochemical processing. This assumption was endorsed by observations demonstrating that ruminal and industrially driven cell wall depolymerization processes share similar underlying biochemical mechanisms [7, 29]. Congruent with these asseverations, under mild thermochemical processing scenarios, Glu-Con correlated negatively (*r* < −0.50) with all lignin-related traits; but associated positively (*r* > 0.4) with characters defining the concentration, extent of glycosylation, and degree of cross-linking of hemicelluloses.

Correspondingly, entries with improved cell wall degradability (both as CWD or Glu-Con) typically displayed a higher absolute release of fermentable glucose (Glu-Rel) upon enzymatic conversion (Fig. 2B). However, while higher bioconversion efficiency (Glu-Con) generally led to a superior release of fermentable glucose, a strictly proportional relationship between the two could not be established. To better illustrate, whereas the “Excellent” and “Cell Wall Mutant” cultivar classes displayed similar bioconversion rates (Fig. 2A), the latter outperformed the former for Glu-Rel across the complete processing series (Fig. 2B). Relative to the “Excellent” class, the class with “Cell Wall Mutant” entries exhibited a higher concentration of cellulose per gram of dry biomass (375 g kg^−1^ dry matter > 358 g kg^−1^ dry matter, *p* < 0.05). Expectedly, since both cultivar classes showed similar levels of cell wall recalcitrance, the “Cell Wall Mutant” class exhibited higher glucose yields upon enzymatic conversion simply because it displayed a superior concentration of cell wall glucose on a dry matter basis. Likewise, because all cultivar groups greatly outranked the “Excellent” class for cellulose content (~406 g kg^−1^ dry matter), these outperformed the latter at the most intensive processing regime where enzymatic convertibility reaches a near-maximum regardless of compositional differences between genotypes.

The amount of glucose released during pretreatment (Glu-Sol) is also an important source of fermentable monosaccharides in biomass-to-ethanol conversion systems. Across the complete processing series, the four distinct entry classes displayed significant differences (*p* < 0.05) in the amount of glucose released in pretreatment liquors (Fig. 2C). These sugars presumably originate from the soluble carbohydrate fraction of the maize stalk, as there was a strong correlation (*r* > 0.7) between the latter and glucose concentration in pretreatment liquors (data now shown). Unlike trends observed earlier, however, glucose yields in pretreatment liquors remained constant across pretreatments of increasing severity, and only exhibited a slight, yet statistically non-significant reduction at the highest processing intensity (Fig. 2C).

### The technical efficiency of cellulosic fuel refineries is influenced by feedstock processing amenability and crop productivity

Techno-economic and life cycle assessments of cellulosic fuel refineries have demonstrated that plant size, commercial viability, and environmental performance are primarily influenced by the extent of fermentable monosaccharides recovered per hectare of harvested feedstock crop [9–14, 30]. This is calculated as the product of the crop’s overall biomass productivity (t ha^−1^) by the total amount of sugars (g kg^−1^ dry matter) released after thermochemical pretreatment and enzymatic saccharification. Given that modeled scenarios do not account for the effect of biomass composition on conversion efficiency, these commonly reiterate that improvements in the productivity of cellulosic fuel refineries can be solely realized through increments in the yield productivity of lignocellulosic crops.

The panel of forage maize cultivars evaluated in this study exhibited highly significant differences (*p* < 0.001) in whole-plant biomass productivity (Table 1). The maximal contrast across entries for biomass yield was approximately 7 t ha^−1^. A closer examination reveals, nevertheless, that differences in biomass yield among the three classes of commercial cultivars were reasonably minor; with the “Poor” index ranking highest (~21 t ha^−1^), followed respectively by the “Excellent” (~20 t ha^−1^) and “Good” (~19 t ha^−1^) digestibility selections. By contrast, differences in total biomass yield between the “Cell Wall Mutant” class (~16 t ha^−1^) and the average of all commercial cultivars (~20 t ha^−1^) were qualitatively more pronounced. The markedly lower yields observed for mutant hybrid varieties were anticipated as numerous studies have demonstrated the detrimental effects on plant fitness conveyed by *bm1* and *bm3* mutations [15].

In general, biomass productivity correlated negatively (*r* ≤ −0.6) with cell wall digestibility and bioconversion properties (CWD, Glu-Con, Glu-Rel; data not shown). From a commercial standpoint, this would tacitly imply that gains in productivity arising from the use of bioenergy feedstocks with improved processing amenability (Glu-Con, Glu-Rel) would be potentially offset by tradeoffs in biomass yield capacity. Consequently, to explore the dynamics of yield-by-quality relations, we have estimated total glucose productivity per hectare (TGP) for all examined entries across all evaluated conditions (Table 4). TGP was calculated as the sum of fermentable glucose recovered in pretreatment (Glu-Sol) and enzymatic saccharification liquors (Glu-Rel) multiplied by stover biomass productivity on a per hectare basis; the latter was estimated from biomass productivity data (Table 1) using the rule-of-thumb assumption that the stover to grain ratio in maize is 1:1 [1].

**Table 4:** Comparison of total glucose productivity (in t ha^−1^) across pretreatments of increasing severity for a panel of commercial silage maize cultivars and experimental cell wall mutants

	TGP (t ha^−1^)			
	Low	Low-mid	Mid-high	High
HYB-001	1.8	2.3	3.1	3.1
HYB-002	2.1	2.9	3.6	3.7
HYB-003	1.6	2.2	2.8	3.4
HYB-004	1.6	2.3	3.1	3.2
HYB-005	1.7	2.5	3.1	3.4
HYB-006	1.5	2.0	3.0	3.1
HYB-007	1.3	1.7	2.5	2.9
HYB-008	1.6	2.1	2.7	3.0
HYB-009	1.3	1.9	3.0	3.0
HYB-010	1.6	2.2	3.2	3.4
HYB-011	1.5	1.8	2.8	2.9
HYB-012	1.2	1.8	2.6	3.2
HYB-013	1.1	1.7	2.4	3.1
HYB-014	1.4	2.2	3.2	3.5
HYB-015	1.4	1.8	2.8	3.3
HYB-016	1.5	2.0	3.0	3.2
HYB-017	1.2	1.9	2.7	3.2
HYB-018	1.2	1.8	2.9	3.2
HYB-010-*bm3*	1.7	2.3	3.0	2.8
HYB-011-*bm3*	1.4	1.9	2.5	2.5
HYB-006-*bm1*	1.2	1.7	2.5	2.6
HYB-009-*bm1*	1.3	1.9	2.8	2.9
HYB-014-*bm1*	1.2	1.8	2.8	2.8

**Fig. 3:**
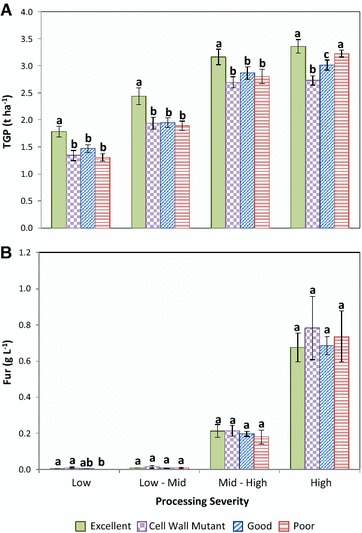
Performance of four distinct cultivar classes (diverging in cell wall digestibility) across pretreatments of increasing severity for **A** TGP and **B** furfural release into pretreatment liquors. Within each processing severity regime, similar *letters* above *bars* indicate non-significant differences according to a Tukey HSD test (*P* = 0.05)

Overall, the four divergent cultivar classes exhibited statistically significant (*p* < 0.05) differences in TGP across all evaluated processing conditions. The “Excellent” cultivar selection consistently outperformed all other cultivar indices; although at the most intensive processing regime, the aforementioned cultivar class did not differ significantly from the “Poor” digestibility class (Fig. 3). Under milder processing regimes, contrasts in TGP among the “Good,” “Poor”, and “Cell Wall Mutant” classifications were statistically non-significant. In principle, these results demonstrate that systematic gains in cell wall degradability (i.e., CWD, Glu-Con, and Glu-Rel) can lead to significant advances in the productivity (TGP) of cellulosic fuel biorefineries, but only under less stringent processing scenarios. Moreover, this is only valid if genetic advances in cell wall degradability properties have not been offset by substantial reductions in biomass yield productivity. For instance, since the “Excellent” and “Poor” cultivar selections exhibited comparatively similar biomass yields (~20 t ha^−1^), the competitive advantage in TGP displayed by the former can be attributed to its improved processing amenability (Glu-Con, Glu-Rel) and higher content of stalk soluble glucose. By contrast, the substantially enhanced bioconversion efficiencies displayed by mutant hybrid varieties (Fig. 2B) were counterbalanced by their greatly inferior biomass productivities (~16 t ha^−1^).

Collectively, these results demonstrate that genetic gains in biomass degradability and processing quality do not necessarily come at the expense of substantial yield reductions. In fact, some of the highest ranked commercial cultivars for cell wall digestibility (i.e., HYB-002 and HYB-003) were also among the highest yielding genotypes in the panel (~21 t ha^−1^). Furthermore, recent investigations have demonstrated that biomass quality, biomass productivity, and grain yield are not mutually antagonistic breeding targets, and can in fact be improved independently [31–34]. The use of interesting cell wall mutations (e.g., *bm3*), however, needs further investigation as it is clear that their introgression in elite material can affect biomass productivity.

### Exploratory cost and environmental impact analysis for the thermochemical processing and enzymatic saccharification of maize feedstocks exhibiting contrasting levels of cell wall degradability

Given that the product value of cellulosic ethanol will be determined by the cost of its manufacturing process, the ultimate goal of the cellulosic fuel industry resides on attaining maximum biomass conversion efficiency at the lowest conceivable processing cost and possible environmental impact. Our conceptual vision explains that this commercial objective can be achieved through the development of bioenergy feedstocks with improved biomass processing amenability. To further explore this vision, we have estimated the environmental and economic benefits that could arise from the wide-scale implementation of cellulosic fuel refineries operating under milder processing conditions. To this end, the environmental performance and cost efficiency of the pretreatment of maize stover was compared in four scenarios (Table 5). For each scenario, all relevant inventory inputs and outputs for the pretreatment process are detailed in Additional file 1: Table S1 (see Additional file 1). Cultivation, harvesting, enzymatic saccharification, and fermentation processes were assumed to be similar under all scenarios analyzed.

**Table 5:** Description of modeled scenarios for the estimation of environmental and cost impacts for a cellulosic ethanol production system using dilute-acid pretreatment

Scenario	Description
I (benchmark scenario)	Pretreatment severity: high Maize feedstock class: “excellent” cell wall digestibility Based on TGP performance (3.5 t ha^−1^) of genotype LG210P106790
II	Pretreatment severity: high Maize feedstock class: “poor” cell wall digestibility Based on the average TGP performance (3.2 t ha^−1^) of de “poor digestibility” cultivar class
III	Pretreatment severity: low-mid Maize feedstock class: “excellent” cell wall digestibility Based on TGP performance (2.9 t ha^−1^) of genotype LG210P106790
IV	Pretreatment severity: low-mid Maize feedstock class: hypothetical scenario Based on TGP performance (~3.7 t ha^−1^) of a hypothetical forage maize cultivar combining the best characteristics available in the entry panel (i.e., highest concentration of stem soluble glucose, holocellulose content, enzymatic convertibility, and biomass yields)

**Fig. 4:**
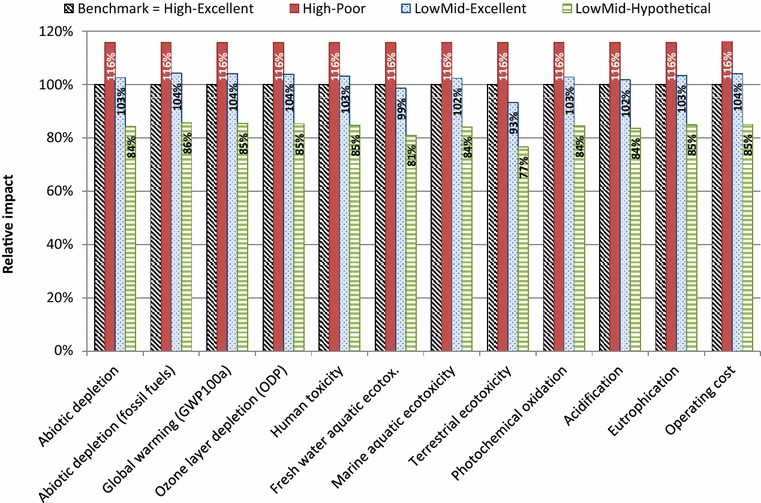
Relative environmental and economic impacts (based on TGP) of scenarios II, III, and IV (refer to Table 4) relative to scenario I (benchmark)

Figure 4 shows the environmental and economic performance (based on TGP) of scenarios II, III, and IV relative to scenario I (benchmark). The environmental and cost impact for the production of cellulosic ethanol from maize cultivars with “Poor” cell wall digestibility (scenario II) was 16 % higher compared to scenario I. By contrast, the environmental and economic performance for processing maize lignocellulose with “Excellent” cell wall digestibility at low-mid severity was comparable with benchmark conditions. This was due to the fact that under low-mid processing severities, chemical and energetic inputs are reduced, but also lower fermentable glucose yields are obtained (2.9 t ha^−1^ compared to 3.7 t ha^−1^). Therefore, there is no significant benefit for the low-mid processing severity in terms of cost or environmental impact. From a commercial standpoint, these manufacturing cost reductions should be accompanied by gains in TGP. At the low-mid processing scenario, the maximum possible TGP (2.9 t ha^−1^; cultivar HYB-002) was just nearly 80 % of the highest TGP achieved within the framework of this investigation (~3.7 t ha^−1^; cultivar HYB-002) (Table 4). We postulate that if breeding would allow for the combination of the best characteristics available in the entry panel (i.e., highest concentration of stem soluble glucose, holocellulose content, enzymatic convertibility, and biomass yields), then maximum TGP at low-mid conditions (~3.7 t ha^−1^) would correspond to 100 % of the highest realizable yields. Under these projections (scenario IV), the cost and environmental impacts of maize pretreatment would be reduced by at least 15 % relative to the benchmark scenario. A closer inspection reveals that terrestrial ecotoxicity would show the greatest improvement (with a relative reduction of 23 % from benchmark conditions), given a significant decrease in heat and NaOH consumption. Improvements in other environmental impact categories (e.g., marine ecotoxicity, global warming potential, abiotic depletion, etc.), were principally attributed to reduced heat inputs. Accordingly, significant cutbacks in chemical and heat inputs in scenario IV led to a 15 % decrement in pretreatment costs; with heat consumption as major contributing factor. In addition to lower acid inputs and a concomitant diminution in alkali usage during slurry neutralization, savings on chemical utilities could also be expected from reductions in cellulase consumption, as several investigations have indicated that bioenergy crops with reduced lignin content typically necessitate lower concentrations of cellulolytic enzymes for their complete and effective fractionation.

### Qualitative analysis of additional benefits for the cellulosic ethanol chain potentially arising from the use of bioenergy feedstocks with improved processing amenability

Other important, albeit less apparent benefits that could arise from the development of feedstocks with reduced lignocellulose recalcitrance correspond to possible tradeoffs in capital investments associated with improvements in pretreatment and downstream processing technologies. To begin with, since highly degradable feedstocks require lower sulfuric acid and temperature usage for their thermochemical fractionation, the industry could potentially move to less costly reactors with lower-corrosion and heat-deformation resistance [35]. Diminished acid consumption during pretreatment also conveys a diminution in salt formation during slurry neutralization. Under scenario IV, salt formation was largely reduced from 0.85 kg kg^−1^ glucose under benchmark conditions to 0.44 kg kg^−1^ glucose. Likewise, Fig. 3B clearly demonstrates that under less severe thermochemical processing regimes, the production of fermentation inhibitors (specifically, furfural) is greatly reduced. In fact, under the low-mid processing scenario, average furfural release (across all cultivar classes) into pretreatment liquors (0.01 g L^−1^) was significantly lower (*p* < 0.0001) than the average furfural release obtained at the highest thermochemical severity (0.72 g L^−1^); the latter considered harmful to the most sensitive yeast strains [36]. Such considerable reductions in salt and fermentation inhibitor formation could lead to cutbacks in the throughput, size, and material costs of downstream equipment, and as such facilitate the integration of consolidated bioprocessing technologies which are expected to greatly reduce both operational and capital costs [10, 12, 35, 37].

The realization of an industry that operates at less severe processing conditions requires that monosaccharide yields recovered from a hectare of established feedstock compete or outperform current realizable productivities. The lingering question remains: how should the industry proceed? Certainly, the development of cellulosic fuel refineries with an improved economic viability and environmental footprint will require an integrative chain approach. In our vision, the development of advanced lignocellulosic feedstocks for the industry will benefit from parallel developments in enzyme and fermentation technologies which maximize the yield and conversion of all fermentable biomass components. In this regard, numerous studies have demonstrated that at mild thermochemical pretreatments, the complementation of cellulolytic cocktails with specialized xylan degrading enzymes greatly improves the release of monomeric xylose and enhances cellulose conversion [36, 38, 39]. Similarly, the derivation of pentoses into added-value ethanologens is seen by experts as a crucial step towards improving the productivity and product value of cellulosic fuels [40–42]. Breeders can simultaneously complement and potentiate these advances by creating cultivars with improved conversion efficiency, higher hemicellulose content, and competitive biomass yields. Small-scale cellulosic biomass refineries are constrained by the poor performance figures on economics and environmental efficiency of biomass-to-fuel conversion technologies, as well as the high costs associated with biomass collection and transportation. Conceivably, if improvements in the productivity and cost performance of biomass-to-fuel conversion systems derived from the use of highly digestible feedstocks can outweigh the high costs of biomass transportation inherent to small cellulosic ethanol biorefineries, it should then be possible to realize projections advocating for small-scale biorefineries and the geographic decentralization of cellulosic ethanol production.

## Conclusions

In this investigation, we demonstrate that systematic changes in cell wall composition leading to improved cell wall digestibility can be advantageous for cellulosic fuel production, especially if “less severe” processing regimes are favored for further development. Exploratory environmental and economic modeling results indicate that the use of maize lignocellulosic feedstocks with improved cell wall degradability can reduce the environmental impact and processing costs of biomass-to-ethanol conversion (i.e., pretreatment and enzymatic saccharification), through lower chemical and heat consumption. Conceptually, if breeding would allow for the combination of the best characteristics available in modern germplasm (i.e., high biomass productivity, high holocellulose content, and improved enzymatic convertibility of cell walls), it should be possible to surpass the productivity of currently available biomass-to-fuel conversion systems using more cost-effective and environmentally sustainable conversion platforms.

## Methods

### Plant materials and field trials

A set of 23 maize hybrids was selected for this investigation (Table 1). Of these, 18 corresponded to forage-dedicated commercial cultivars bred primarily for Northern-European markets. These cultivars were selected to be diverse for ruminal cell wall digestibility and overall biomass productivity. The panel also included five experimental hybrids (derived from five of the aforementioned commercial cultivars) carrying either the brown-midrib *3* (*bm3*) or a Biogemma proprietary brown-midrib 1 (*bm1*) mutation [17].

Entries were evaluated in replicated trials (in adjacent completely randomized blocks) at three different locations in The Netherlands (Biddinghuizen, Eindhoven, and Wouw) during the summer of 2012. However, due to unfavorable climatic conditions that year, the complete panel was only successfully grown at Eindhoven. Accordingly, the trial at Biddinghuizen included only the commercial cultivars, and the trial at Wouw included only the experimental mutants. In all trials, genotypes were planted in two-row plots with a length of 2.5 m and an inter-row distance of 0.75 m at a density of ten plants m^−1^. For each plot, stalks of ten randomly selected plants were harvested at a 10 cm stubble height at silage maturity. At this physiological stage, differences between genotypes in stem cell wall composition and digestibility were expected to be largely genetic [43]. Due to the intensive workload, locations were harvested on separate days. Collected biomass feedstocks were chopped and air dried at 70 °C for 48 h, and were subsequently ground through a 1 mm screen using a hammer mill. For both, cell wall compositional analyses and bioconversion assays, feedstock samples were produced by pooling, per genotype, the milled material collected from all experimental plots as to minimize random variation due to environment and processing (as would occur in the industry).

### Compositional analysis

All biomass and cell wall compositional analyses, with the exception of the degree of substitution of hemicellulose and cell wall glucose concentration, were estimated using near infrared reflectance spectroscopy at Limagrain Nederland B.V. Briefly, ground stover samples were scanned using a FOSS NIRS DS2500 system (Foss, Hillerod, Denmark) and biochemical predictions were obtained using calibration equations developed at INRA Lusignan [44]. This calibration is specific for the analysis of maize stem forage quality and cell wall compositional traits (including detergent fiber components) and ruminal cell wall digestibility parameters. A detailed description of all evaluated traits is presented in Table 6 and calibration statistics are presented in Additional file 2: Table S2 (see Additional file 2).

**Table 6:** Description of quality traits measured on stem material of 23 maize silage hybrids diverging in cell wall digestibility

Trait	Unit	Description
CW	g kg^−1^ DM	Stem cell wall content; determined as neutral detergent fiber (NDF)
Cel	g kg^−1^ DM	Stem cellulose content; determined as the difference between acid detergent fiber (ADF) and acid insoluble lignin (ADL)
Lig	g kg^−1^ DM	Stem acid insoluble lignin content; determined as ADL
Cel/CW	g kg^−1^ CW	Stem cellulose content relative to cell wall content (CW)
Hem/CW	g kg^−1^ CW	Stem hemicellulose content relative to cell wall content (CW)
Lig/CW	g kg^−1^ CW	Stem acid insoluble lignin content relative to cell wall content (CW)
pCa I	g kg^−1^ CW	Esterified p-coumaric acid released after alkaline hydrolysis of the cell wall at 25 °C
pCa II	g kg^−1^ CW	Total p-coumaric acid released after alkaline hydrolysis of the cell wall at 170 °C
FA I	g kg^−1^ CW	Esterified ferulic acid released after alkaline hydrolysis of the cell wall at 25 °C
FA II	g kg^−1^ CW	Total ferulic acid released after alkaline hydrolysis of the cell wall at 170 °C
Di-FA I	g kg^−1^ CW	Esterified di-ferulic acid released after alkaline hydrolysis of the cell wall at 25 °C
Di-FA II	g kg^−1^ CW	Total di-ferulic acid released after alkaline hydrolysis of the cell wall at 170 °C
DHS	_%_	Degree of hemicellulose substitution, expressed as the ratio of cell wall arabinose to cell wall xylose (Ara/Xyl)
H	g kg^−1^ CW	H lignin content estimated as 4-*p*-hydroxybenzaldehyde released following nitrobenzene oxidation of the cell wall at 170 °C
S	g kg^−1^ CW	S lignin content estimated as syringylaldehyde released following nitrobenzene oxidation of the cell wall at 170 °C
G	g kg^−1^ CW	G lignin content estimated as vanillin released following nitrobenzene oxidation of the cell wall at 170 °C
Glu-Sol	g kg^−1^ DM	Amount of glucose released from 1 g of dry biomass into pretreatment liquors following thermochemical pretreatment
Glu-Rel	g kg^−1^ DM	Amount of glucose released from 1 g of dry biomass after pretreatment and enzymatic saccharification
Glu-Con	% CW glucose	Percentage of total cell wall glucose released after pretreatment and enzymatic saccharification
CWD	% NDF	In vitro ruminal cell wall digestibility; determined as the difference in NDF content before and after sample incubation in rumen liquor for 48 h relative to NDF content prior to incubation

The degree of hemicellulose substitution, measured as the ratio of cell wall arabinose-to-xylose, was derived from the analysis of cell wall neutral sugar components; the latter was determined by gas chromatography essentially as described by Englyst and Cummings [45]. Briefly, lyophilized water un-extractable solids were first treated with 72 % sulfuric acid (1 h, 30 °C), followed by a second hydrolysis process with 1 M sulfuric acid (3 h, 100 °C). Released neutral sugars were then derivatized to their respective alditol isoforms and quantified on an Agilent 7890A Gas Chromatography System (Agilent Technologies, Santa Clara, CA) using a DB-250 column (Agilent Technologies, Santa Clara, CA).

### Bioconversion efficiency

#### Thermochemical pretreatment and enzymatic conversion efficiency

Thermal dilute-acid pretreatments of increasing severity were performed in triplicate on all ground maize stalk samples (Table 7). Reactions were carried out using 25 mL custom-built stainless steel high-pressure reactors equipped with a K-type thermocouple and a 12 cm stainless steel thermocouple probe. Biomass samples (500 mg) were contained inside heat/acid-resistant nylon filter bags (ANKOM Technology Corporation, Fairpoint, NY) which allowed for easy biomass transfer while preventing biomass losses during processing reactions. During pretreatments, two separately controlled oil baths were employed; the first one—set at 180 °C—was used to rapidly heat up reactors, while the second bath was used to control reactions at the desired temperature. Depending on the conditions, target temperatures were typically reached between 3 and 5 min. To maintain the temperature within ±1.0 °C of the target temperature, reactors were either manually hoisted from the oil bath or re-submerged in the higher temperature oil bath when necessary. After the desired treatment time, reactions were rapidly quenched by plunging the reactors in an ice-water bath. Pretreatment liquors were collected for further chemical analyses, and biomass samples were washed with abundant distilled water.

**Table 7:** Thermochemical parameters used for the pretreatment of stem material of 23 maize silage hybrids diverging in cell wall digestibility

Processing severity	Temperature (°C)	Duration (min)	Acid loading^a^ (%)	Solids loading^b^ (%)
Low	150	30	0.07	3.3
Low-mid	150	20	0.17	3.3
Mid-high	175	10	0.17	3.3
High	180	10	0.34	3.3

^a^98 % H_2_SO_4_ (*w/v* %)

^b^Pretreatment-slurry solids to liquid ratio (*w/v* %)

#### Analysis of pretreatment liquors

After thermal dilute-acid pretreatment, pretreatment liquors were filtered through a 0.45 µm syringe filter. Monomeric glucose release (Glu-Sol) was analyzed using a Dionex High Pressure Liquid Chromatography system (Dionex, Sunnyvale, CA) equipped with a CarboPac Pa100 column (Dionex, Sunnyvale, CA). Furfural and 5-(hydroxymethyl)furfural concentrations were analyzed using a Waters HPLC–PDA (Waters Associates, Milford, MA) equipped with an Altima HP C18 (5 µm) column (Alltech, Deerfield, IL).

#### Enzymatic saccharification

Bioconversion efficiency was analyzed following the National Renewable Energy Laboratory Analytical Procedure-009 [46]. Pretreated samples contained within nylon filter bags were treated with 250 µL of an Accelerase 1500 cellulolytic enzyme cocktail (Genencor B.V., Leiden, NL) in 40 mL 0.1 M citrate buffer. The enzyme load provided 50 filter paper units of cellulase per gram cellulose. Samples were then incubated at 50 °C in an Innova 42 air incubator (New Brunswick Scientific, Enfield, CT) at 200 RPM for 24 h. Enzymatic saccharification liquors were analyzed for glucose content using a Boehringer Mannheim d-Glucose kit (Boehringer Mannheim, Indianapolis, IN, USA). The colorimetric assay was adapted to a 96 micro-titer plate format, and spectrophotometric reads were made using a Bio-Rad 550 Micro-plate Reader (Bio-Rad, Richmond, CA). For all samples, glucose content was expressed as both, the amount of glucose released from 1 g of dry biomass (Glu-Rel) and the percentage of total cell wall glucose released after enzymatic saccharification (Glu-Con) (Table 7).

### Statistical analyses

One-way analysis of variance (ANOVA) was used to determine the significance of differences between genotypes (i.e., commercial cultivars and experimental hybrids) for whole-plant biomass yield, CWD, stem fiber components, and cell wall characteristics. For a subset of five commercial hybrids and their mutant counterparts, Tukey HSD analysis (*P* = 0.05) was used to conduct targeted pairwise comparisons between hybrids and their corresponding mutant. For bioconversion parameters (Glu-Rel, Glu-Con, Glu-Sol, Furfural release in pretreatment liquors), two-way ANOVA (using “genotype” and “pretreatment severity” as factors) was used to evaluate the significance of differences between genotypes and processing regimes. General ANOVA, followed by a Tukey HSD post hoc test (*P* = 0.05), was used to establish the statistical significance of differences for bioconversion traits and TGP among “digestibility classes” (Table 1). Pearson correlations between bioconversion parameters (and CWD) and stem fiber and cell wall components were also independently determined for each pretreatment condition analyzed. All statistical analyses were performed using the GenStat for Windows 14th Edition Software Package (VSN International, Hemel Hempstead, UK).

### Environmental and economic analyses

#### Scope

**Fig. 5:**
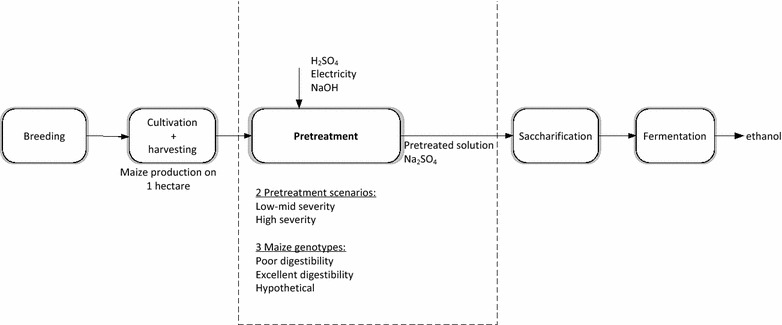
Flow scheme for the production of cellulosic ethanol from maize lignocellulosic biomass using dilute-acid pretreatment; *dotted lines* delimit the *system boundary*. During cellulosic ethanol production, the polysaccharide fraction (cellulose and hemicellulose) of plant lignocellulose is deconstructed via thermochemical pretreatment. The resulting slurry is neutralized to prevent enzyme limitations during the saccharification stage. Fermentation of saccharification broths yields ethanol

A schematic overview of the production chain for maize cellulosic ethanol is presented in Fig. 5. For this study, explorative environmental and economic impact calculations focused on the pretreatment of maize lignocellulosic biomass (i.e., system boundary) under various biomass and processing scenarios (Table 5). The results were compared relative to a benchmark scenario, to indicate the most promising scenario for future research. The major variables considered were the consumption of energetic (heat) and chemical inputs (sulfuric acid, and sodium hydroxide [NaOH] used for neutralizing pretreatment slurries). The analysis concentrated on processing conditions and their subsequent impact, and not on equipment cost or infrastructure requirements. Materials and energy consumption associated with the establishment of infrastructure have been excluded. For modeling the upstream secondary processes, Ecoinvent Life Cycle Inventory data were used [47]. Crop cultivation and harvesting conditions were identical for all scenarios analyzed (Table 5) and were therefore not included in the analysis. Similar assumptions were held for the enzymatic saccharification and fermentation processes. During enzymatic saccharification, an overdose of cellulase was applied to prevent incomplete biomass conversion due to insufficient enzyme loading; thus the performance of this process did not vary between analyzed scenarios (Table 5). Total glucose productivity per hectare of maize production (recovered following pretreatment and enzymatic saccharification) was used as the functional unit for assessing the environmental and economic performance of the maize stover pretreatment.

### Mass and energy balances and impact calculations

Sulfuric acid loadings, maize biomass yields, and fermentable glucose productivity were obtained from the empirical data described in this study. For all scenarios analyzed, sodium hydroxide (NaOH) dosage and sodium sulfate (Na_2_SO_4_) production were calculated stochiometrically based on sulfuric acid loadings (Eqs. 1, 2, respectively). The energy demand required for heating the solution during pretreatment was calculated based on the heat capacity of the solution (Eq. 3). These inputs were basis to determine upstream life cycle impacts. Costs for chemical inputs were estimated to be 0.035 $/kg H_2_SO_4_, 0.45 $/kg NaOH (based on 2010 prices as given in [48]), and 0.11 $/kWh electricity (industrial OECD price of 2010, based on IEA/OECD Energy prices and taxes). For heat production, an upstream electricity demand of 1.05 kWh upstream/kWh heat was applied [49]. All inputs and outputs were assumed to a production scale capacity, and were used to estimate the economic performance of the production system.

1 $$m_{{{\text{NaOH}}}} \left( {{\text{kg}}} \right) = {\text{ }}2m_{{{\text{H}}_{2} {\text{SO}}_{4} }} {{{\text{MW}}_{{{\text{NaOH}}}} } \mathord{\left/ {\vphantom {{{\text{MW}}_{{{\text{NaOH}}}} } {{\text{MW}}_{{{\text{H}}_{2} {\text{SO}}_{4} }} }}} \right. \kern-\nulldelimiterspace} {{\text{MW}}_{{{\text{H}}_{2} {\text{SO}}_{4} }} }}$$

2 $$m_{{{\text{Na}}_{{\text{2}}} {\text{SO}}_{{\text{4}}} }} \left( {{\text{kg}}} \right) = {\text{ }}m_{{{\text{H}}_{{\text{2}}} {\text{SO}}_{{\text{4}}} }} {{{\text{MW}}_{{{\text{Na}}_{2} {\text{SO}}_{4} }} } \mathord{\left/ {\vphantom {{{\text{MW}}_{{{\text{Na}}_{2} {\text{SO}}_{4} }} } {{\text{MW}}_{{{\text{H}}_{2} {\text{SO}}_{4} }} }}} \right. \kern-\nulldelimiterspace} {{\text{MW}}_{{{\text{H}}_{2} {\text{SO}}_{4} }} }}$$

3 $$E_{\rm{heat}} \left( {\rm{kWh}} \right) = {\rm{cp}}_{\rm{water}} \left( {m_{\rm{biomass}} + m_{{{\rm{H}}_{ 2} {\rm{SO}}_{ 4} }} + m_{\rm{NaOH}} + m_{\rm{water}} } \right)\frac{{\left( {T_{\rm{pretreatment}} - T_{\rm{room}} } \right)}}{\eta } \times 3.6\times10^{ - 6}$$

Environmental impact calculations were assessed using the life cycle analysis software SimaPro Version 7.3.3 (PRé Consultants, The Netherlands) in conjunction with the CML method (CML-IA Baseline V3.01/EU25). The following inputs were selected in SimaPro:

- Sulfuric acid (98 % H_2_SO_4_), at plant/RER Mass
- Water decarbonized ETH—S
- Sodium hydroxide, without water, in 50 % solution state {GLO}| market for | Alloc Def,—S
- Heat industrial furnace > 100 kW—S

All inputs of the pretreatment were utilized to estimate the cost performance. All cost and environmental impacts of the pretreatment are allocated for each scenario to the total glucose yield.

## Additional files

10.1186/s13068-016-0479-0 Inventory of chemical and energetic inputs and outputs for the pretreatment of maize biomass under four different scenarios.
10.1186/s13068-016-0479-0 Near infrared reflectance spectroscopy calibration statistics for maize stover cell wall composition traits.

## Abbreviations

ANOVA: analysis of variance
*bm1*: biogemma proprietary brown-midrib 1 mutation
*bm3*: brown-midrib 3
CWD: in vitro cell wall digestibility
Glu-Con: percentage of total cell wall glucose released after pretreatment and enzymatic saccharification
Glu-Rel: amount of glucose released from 1 g of dry biomass after pretreatment and enzymatic saccharification
Glu-Sol: amount of glucose released from 1 g of dry biomass into pretreatment liquors following thermochemical pretreatment
Na_2_SO_4_: sodium sulfate
NaOH: sodium hydroxide
TGP: total glucose productivity per hectare
