# Supplementary material for: Maize feedstocks with improved digestibility reduce the costs and environmental impacts of biomass pretreatment and saccharification
Source: Biotechnol Biofuels. 2016 Mar 15;9:63. doi: 10.1186/s13068-016-0479-0 (PMC4791978; doi:10.1186/s13068-016-0479-0)
Supplement: Supplementary file 1 — 10.1186/s13068-016-0479-0 Inventory of chemical and energetic inputs and outputs for the pretreatment of maize biomass under four different scenarios. [file 13068_2016_479_MOESM1_ESM.pdf]

Table S1. Inventory of chemical and energetic inputs and outputs for the pretreatment of maize biomass under four different scenarios.

	Inputs				Outputs	
Scenario	Biomass (kg)	H_2SO_4 (kg)	NaOH (kg)	Heat (kWh)	Total Glucose Production (kg)	Na_2SO_4 (kg)
I	$10.5*10^3$	2164	44.1	$149*10^3$	3700	3133
II	$10.5*10^3$	2164	44.1	$149*10^3$	3200	3133
III	$10.5*10^3$	1082	22.1	$121*10^3$	2900	1567
IV	$11.0*10^3$	2267	46.2	$156*10^3$	3700	3283
