# Supplementary material for: Maize feedstocks with improved digestibility reduce the costs and environmental impacts of biomass pretreatment and saccharification
Source: Biotechnol Biofuels. 2016 Mar 15;9:63. doi: 10.1186/s13068-016-0479-0 (PMC4791978; doi:10.1186/s13068-016-0479-0)
Supplement: Supplementary file 2 — 10.1186/s13068-016-0479-0 Near infrared reflectance spectroscopy calibration statistics for maize stover cell wall composition traits. [file 13068_2016_479_MOESM2_ESM.pdf]

Table S2. Near infrared reflectance spectroscopy calibration statistics for maize stover cell wall composition traits.

Trait	Unit	*N* [a]	Mean	Min.	Max.	R^2	SEC	SECV
CW	$g\ Kg^{-1}$ DM	710	61.37	40.73	82.00	0.93	1.81	1.84
ADF [b]	$g\ Kg^{-1}$ DM	357	36.16	20.12	52.19	0.94	1.34	1.39
Lig	$g\ Kg^{-1}$ DM	337	3.96	0.58	7.35	0.59	0.73	0.76
pCa I	$g\ Kg^{-1}$ CW	1563	15.60	1.45	21.72	0.87	1.23	1.26
pCa II	$g\ Kg^{-1}$ CW	1494	11.54	2.24	20.83	0.88	1.09	1.13
FA I	$g\ Kg^{-1}$ CW	1561	5.55	3.01	8.08	0.64	0.51	0.52
FA II	$g\ Kg^{-1}$ CW	1499	6.90	4.21	9.60	0.66	0.53	0.54
Di-FA I	$g\ Kg^{-1}$ CW	516	0.15	0.03	0.27	0.66	0.02	0.03
Di-FA II	$g\ Kg^{-1}$ CW	514	0.33	0.11	0.54	0.61	0.04	0.05
H	$g\ Kg^{-1}$ CW	1048	1.43	0.00	3.10	0.74	0.28	0.30
S	$g\ Kg^{-1}$ CW	1054	7.22	0.41	14.03	0.79	1.03	1.09
G	$g\ Kg^{-1}$ CW	1051	6.38	1.30	11.46	0.60	1.07	1.11

[a] *N* is the number of samples analyzed for development of calibration equation; R^2 is the coefficient of determination between laboratory analysis and NIRS prediction, SEC is the standard error of calibration and SECV is the standard error of cross-validation predictions.

[b] Acid detergent fiber
